# Prevalence of enterotoxins and other virulence genes of *Staphylococcus aureus* caused subclinical mastitis in dairy cows

**DOI:** 10.14202/vetworld.2020.1193-1198

**Published:** 2020-06-26

**Authors:** Rania M. Ewida, Amira A. T. Al-Hosary

**Affiliations:** 1 Department of Food Hygiene (Milk Hygiene), Faculty of Veterinary Medicine, New Valley University, New Valley 72511, Egypt; 2 Department of Animal Medicine (Infectious Diseases), Faculty of Veterinary Medicine, Assiut University, Assiut 71526, Egypt

**Keywords:** enterotoxins, *mecA*, *nuc*, polymerase chain reaction, *Staphylococcus aureus*, subclinical mastitis

## Abstract

**Background and Aim:**

Milk production is one of the main props for the national economy. One of the crucial problems in this industry is subclinical mastitis, which harms this industry that considered the backbone of the economy. It is an infectious and zoonotic disease; the infection can spread between dairy animals through milkers’ hands, and milking machines, while the human infection occurs due to the consumption of apparently hygienic milk. *Staphylococcus aureus* is one of the main causative agents of clinical and subclinical mastitis. It is also considered one of the bacteria incriminated in food intoxication of humans due to its virulence factors as enterotoxins and toxic shock syndrome. The current study was designed to assess the prevalence of *S. aureus* and its enterotoxins, as well as, its other virulence factors in milk collected from cows that suffer from subclinical mastitis.

**Materials and Methods:**

Sixty cows were collected from different dairy farms located in Assiut Governorate, Egypt. These cows were subjected to the clinical examination of the udder and its lymph nodes before sampling. Milk samples were collected from clinically healthy udders. All the milk samples were examined by California mastitis test (CMT), polymerase chain reaction (PCR), and enzyme-linked immunosorbent assay (ELISA) for confirmation subclinical mastitis, presence of *S. aureus* and its enterotoxins genes and other virulence factors in the examined milk samples.

**Results:**

The cows included in the current study had healthy udders. The sixty collected milk samples were tested by CMT. 48/60 (80.0%) were positive samples; from the 48 positive samples, 46 (95.83%) samples were confirmed positive by *S. aureus* *16s rRNA* PCR assay. Multiplex PCRs confirmed the presence of staphylococcus enterotoxin gene C (*sec*) in one sample, staphylococcus enterotoxin gene D (*sed*) in 23 samples, while ELISA assay confirmed the presence of the same enterotoxin in only two samples. On the other hand, other groups of genes responsible for some other virulence factors of *S. aureus* like the extracellular thermostable nuclease (*nuc*) gene were found in 33 samples, while toxic shock syndrome (*tsst*) gene and methicillin restraint *S. aureus* (*mecA*) gene were not detected in this study.

**Conclusion:**

Subclinical mastitis is one of the hidden factors that adversely affect the health of both animals and humans. The milk is usually appeared good and may be consumed by humans especially children; however, it causes severe public health problems. In addition, the infected animals with this form of mastitis can spread the infection to other dairy animals and may be turned to a clinical case of contagious mastitis that may be ended by animal culling or death. *S. aureus* is one of the main causes of subclinical mastitis in cattle. In addition to extracellular thermostable nuclease (*nuc*) gene, staphylococcus enterotoxin gene C (*sec*) and staphylococcus enterotoxin gene D (*sed*) are the most common virulence genes confirmed in subclinical mastitis milk. These results highlighted the need to apply more hygienic measures in the dairy farms to avoid spreading the infection between animals to ensure the production of safe and healthy food to humans.

## Introduction

Mastitis is one of the most common infectious diseases affecting dairy animals. It is considered the most economical disease of the dairy industry worldwide, including Egypt [1,2]. Mastitis, especially subclinical mastitis, is responsible for severe economic losses in the form of reduction of milk production, low-quality milk, costs of treatment, and veterinary service, besides, the increase in the culling rate [3]. Mastitis is one of the multifactorial diseases that usually resulting due to synergistic action between some risk factors that enhance the action of the mastitis pathogens. Bacteria are considered the major cause of bovine mastitis. It is responsible for both environmental mastitis caused by *Escherichia coli* and contagious mastitis caused by *Staphylococcus* spp. and *Streptococcus* spp. [4].

*Staphylococcus aureus* is the main cause of mastitis as well as it is the second identified bacteria causing food poisoning in humans through its ability to produce several enterotoxins in milk and milk products. These enterotoxins include SEA to SEE and SEG to SEQ. The previous studies confirmed that 25% of food poisoning outbreaks usually are caused by classical enterotoxins (SEA to SEE) [5]. According to the official European Union data from 2011, 346 foodborne outbreaks were attributed to *Staphylococcus* spp. This represented 6.4 of all reported outbreaks. Furthermore, *S. aureus* can produce other virulence factors such as exfoliative toxin A and B and toxic shock syndrome (TSST-1) [6,7]. Although, pasteurization can destroy *S. aureus* but cannot affect their enterotoxins which are transmitted to humans and cause a public health hazard Intramammary and systemic administrations are common methods for the treatment of mastitis around the world. The long history of using antibiotics in the dairy farms to treat infectious diseases, especially mastitis and this un-responsible application lead to developing the resistance of bacteria to the antibiotics. Methicillin-resistant *S. aureus* (MRSA) has been characterized by the presence of *mecA* gene, which can resist the b-lactam antibiotics [8,9]. MRSA was isolated from milk samples in Egypt in two different localities, including Assiut and El-Mansoura governorates [10-12].

The present study was planned to estimate the prevalence of *S. aureus* and its enterotoxins’ genes in bulk milk samples from cows infected with subclinical mastitis using California mastitis test (CMT), molecular, and serological assays.

## Materials and Methods

### Ethical approval

Sampling and lab work were followed the ethical guidelines and principles by both Assiut University and veterinary authorities in Assiut Governorate for scientific research involving animals.

### Study area, study period and samples collection

Milk samples were collected from different cows’ farms located in Assiut Governorate, Egypt.

Assiut is the biggest governorate in Upper Egypt and it is the capital of Upper Egypt [13]. The collection of samples and samples analysis were done from April to September 2019.

A total of 60 bulk milk samples were collected from 60 cows. The samples were collected in clean, dry, and sterile 50 ml screw-capped falcon tubes, under aseptic conditions after cleaning and disinfection of the udder. Each sample was tested individually using CMT then divided into two parts, each one stored at −20°C until DNA extraction and enzyme-linked immunosorbent assay (ELISA) analysis.

### Clinical examination

All the cows’ udders were clinically examined before sampling according to previously described methods for the examination of the udder and its regional lymph nodes [14].

### CMT

Milk samples were subjected to CMT as a screening test for subclinical mastitis. An equal amount of milk and CMT solution was gently mixed for 10 s in CMT plastic plate, and then the result was recorded by a single test reader. Positive milk samples in CMT were examined for confirmation of *S. aureus* infection and its enterotoxins genes by molecular assays [15].

### Polymerase chain reaction (PCR)

#### DNA extraction

DNA was extracted from positive CMT bulk milk samples using Qiagen DNA Blood Mini kit (Cat. No. 51104, Hilden, Germany) according to manufacturer instruction, and then the extracted DNA was stored at –20°C.

#### DNA amplification

The *S. aureus* and its enterotoxin genes were tested using different primers for *16S rRNA*, *sea-see*, *tsst, nuc*, and *mec A* (Table-1) [16-21]. PCR reactions were performed in final volume 25 μl including 12.5 μl of 2× PCR master mix (Green Master, Promega, USA), 150 ng of DNA template, 1 μl of each primer, and 5.5 μl of Nuclease-free water. Multiplex PCR was performed for the detection of *sea-sec* genes (reaction A) and *sed-see* genes (reaction B) in final volume 25 μl including 12 μl of Multiplex PCR master mix (Qiagen, German), 150 ng of DNA template, and 1 μl of each primer and Nuclease-free water was added until the volume was completed to 25 μl. The amplification was performed in Gradient Thermal Cycler (Veriti Applied Biosystem, USA). The thermal profile for all genes was 94°C for 5 min, followed by 35-40 cycles of denaturation at 94°C for 50 s., and the annealing temperatures were set according to each primer (s) (Table-1) followed by extension at 72°C for 1 min and the final extension at 72°C for 10 min then PCR products were preserved at 4°C.

**Table-1:** Primer sets used to amplify fragments of *16S rRNA*, enterotoxins, and virulence factors genes in *S. aureus.*

Primer Name	Sequence (5’-3’)	Amplified product size (bp)	Annealing Temperature	No. of cycles	Reference
*16S rRNA*	GTA GGT GGC AAG CGT TAT CC CGC ACA TCA GCG TCA G	228	50°C/50 s	40 cycles	[16]
*sea*	GCA GGG AAC AGC TTT AGG C GTT CTG TAG AAG TAT GAA ACA CG	521	48°C/1 min 44°C/1 min (reaction A)	15 cycles	
*seb*	ACA TGT AAT TTT GAT ATT CGC ACT G TGC AGG CAT CAT GTC ATA CCA	667		20 cycles	[17]
*sec*	CTT GTA TGT ATG GAG GAA TAA CAA TGC AGG CAT CAT ATC ATA CCA	284			[16]
*sed*	CCA ATA ATA GGA GAA AAT AAA AG ATT GGT ATT TTT TTT CGT TC	278	57°C/2 min (reaction B)	35 cycles	[18]
*see*	AGG TTT TTT CAC AGG TCA TCC CTT TTT TTT CTT CGG TCA ATC	209			[19]
*tsst*	GCT TGC GAC AAC TGC TAC AG TGG ATC CGT CAT TCA TTG TTA T	559	50°C/50 s	40 cycles	[16]
*nuc*	GCG ATT GAT GGT GAT ACG GTT AGC CAA GCC TTG ACG AAC TAA AGC	270	of 50°C/1 min	40 cycles	[20]
*mecA*	AAA ATC GAT GGT AAA GGT TGG C ATC TGT ACT GGG TTA ATC	533	of 50°C/1 min	40 cycles	[21]

S. aureus: Staphylococcus aureus

### Gel electrophoresis

PCRs products were electrophoresed in 1% agarose gel (GX 040.90, Gen Agarose, L.E., Standard DNA/RNA agarose, Molecular Biology Grade, Inno-Train Diagnostic, D–61476, Kronberg/Taunus) containing 10% diluted ethidium bromide 1 µl/ml electrophoresis buffer at 100 volts for 60 min. Using 100 bp DNA–ladder in SCiE–PLAS, HU 10, 5636, UK. Then, the gel was visualized by the high-performance ultraviolet (UV) transilluminator (UV, INC, UK) and DOC–It ® LS, Image acquisition–software (UVP, INC, UK).

### ELISA

The milk samples were prepared and tested as described in the manual kit of RIDASCREEN® SET (Art. No.: R4101). It is a sandwich ELISA used for the detection and the identification of staphylococcal enterotoxins A, B, C, D, and E in milk. The test was performed according to the manufacturer’s instructions.

### Statistical analysis

The prevalence of *S. aureus* as a cause of the subclinical mastitis, its virulence enterotoxins, and their statistical significance was calculated using Chi-square test (SPSS, Chicago, USA). Any difference was considered significant at 5% threshold values [22].

## Results

**Figure-1:**
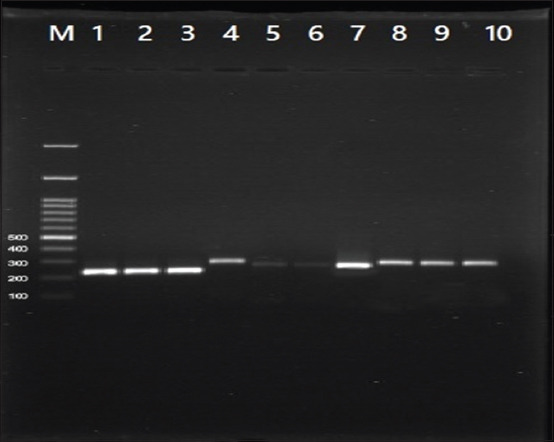
Amplicons of the genes *16S rRNA*, *sec*, *sed*, and *nuc* gene. Lane (M) DNA ladder 100 bp; lanes (1-3) positive samples of *16S rRNA* gene at 228 bp; lane (4) positive sample of *sec* gene at 284 bp, lanes (5-7) positive samples of *sed* gene at 278 bp and lanes (8-10) positive samples *nuc* gene at 270 bp.

The udders of the sixty cows were clinically examined, and all were apparently healthy, and the related cranial and caudal supra-mammary lymph nodes were normal in size and constituent. Forty-eight (80%) milk samples were positive using CMT out of the sixty collected cow’s bulk milk samples. *16S rRNA* PCR confirmed the presence of *S. aureus* in 46/48 (95.83%) samples, which were CMT positive (Table-2). The PCR assays confirmed the presence of *sec* gene (C enterotoxin) only in one sample (2.17%), the *sed* gene (D enterotoxin) was found in 23 (50%) samples. In addition, the occurrence of virulence genes (*nuc*) was confirmed in 33 (71.73%) of the examined milk samples (Figure-1). The statistical analysis revealed highly significant differences between the tested enterotoxin genes and virulence genes with p=0.00001. ELISA assay confirmed the occurrence of enterotoxin D in only two samples (4.34%) (Table-3).

**Table-2:** Prevalence of subclinical mastitis in cow’s milk samples (n=60) caused by *S. aureus.*

Results	CMT		*16S rRNA* gene of *Staphylococcus aureus*	
	
	Positive/Total	%	Positive/Total	%
+ ve	48/60	80	46/48	95.83
- ve	12/60	20	2/48	4.17

*S. aureus: Staphylococcus aureus*, CMT: California mastitis test

**Table-3:** Frequency distribution of some genes of enterotoxins and virulence factors of *S. aureus* occurred in bovine subclinical mastitic milk.

Genes of enterotoxins and virulence factors	PCR assay			ELISA assay	
	
	Positive/Total	%	p-value	Positive/Total	%
C	1/46	2.17	0.00001	0/46	0
D	23/46	50	0.00001	2/46	4.34
*nuc*	33/46	71.73	0.00001	0/46	0

The result is significant at p<0.05.*S. aureus: Staphylococcus aureus*, PCR: Polymerase chain reaction, ELISA: Enzyme-linked immunosorbent assay

## Discussion

Subclinical mastitis is a trigger factor for contagious mastitis in dairy farms and considered one of the most important diseases that affect dairy animals. Clinical mastitis is characterized by signs of inflammation on the udder associated with physical, chemical, cellular, and bacteriological changes in milk in addition to systemic signals as fever, anorexia, lameness, and toxemia. Contrariwise, the subclinical mastitis produces no clinical signs, but it produces cellular changes in the mastitis milk. All the examined cows showed no clinical signs of mastitis with apparently healthy udders. This finding came in agreement with the results of some previous studies which confirmed the subclinical mastitis using CMT in cows with apparently healthy udder [23]. Out of the examined milk samples, 80% were positive for the subclinical mastitis through the results of CMT. This finding confirmed that subclinical mastitis is more hazardous than the clinical mastitis because it is a hidden form of the infection and the udder and milk are normal without any physical changes, only the somatic cell count (SCC) increases more than normal count which is 100,000: 200,000 cell/ml. This SCC includes both the bacterial cell responsible for mastitis like *S. aureus* and the immune cells responsible for defense. These animals act as a potential source for infection of other healthy susceptible animals. This finding came in agreement with the previous studies; however, the result in this study is slightly higher than the results found in Ismailia Province, Egypt; Musanze district, Rwanda; India and Ethiopia, where the subclinical mastitis was confirmed in 71.6%, 60%, 48.14%, and 36.67% of the examined milk samples, respectively [24-27]. *S. aureus* is one of the relevant and important major mastitis pathogens and responsible for food intoxication transmitted to humans through the consumption of contaminated milk. The current study confirmed that 95.83% of the CMT examined bovine bulk milk samples were positive for *S. aureus* subclinical mastitis. This incidence was in agreement with some previous studies in Ethiopia and Egypt, where the authors confirmed that *S. aureus* was a predominant pathogen confirmed in subclinical mastitis cases [27-29]. Moreover, the obtained result was higher than the previous finding confirmed in the previous study in Benha Province, Dakahlia Province, and West Bank-Palestine. In these studies, the authors confirmed the presence of *S. aureus* in 90.4%, 81.3%, and 68.4% of the examined milk samples, respectively [30-32]. This high incidence of *S. aureus* may be attributed to several factors: (1) Its ability to be hidden inside the udder especially with poor hygienic measures before and after milking; (2) the neglected periodical cleaning; (3) the disinfection of milking pens, milking machine, and/or millker’s hands which act as a potential vehicle that can carry the infection from animal to another; and (4) the neglected periodical milk examination using direct and indirect methods that can detect any increase in the SCC that acts as an indicator for subclinical mastitis. On the other hand, the less effective or insufficient veterinary services and irresponsible or insufficient use of antimicrobial drugs in cases of clinical mastitis may lead to the occurrence of antimicrobial-resistant strains of *S. aureus*. This leads to the formation of botryomycosis, which is one of the most serious complications and residues of *S. aureus* infection in the udder. In these cases, the bacteria become protected inside the udder tissue and resist the treatment. Furthermore, these animals will become a potential source of the infections even with no clinical signs observed and this makes *S. aureus* one of the major contagious subclinical mastitis pathogens all over the world [33]. In addition, the genes of the *S. aureus* enterotoxins were detected using two different techniques include molecular and serological assays (PCR and ELISA). The enterotoxin D was found in 50% and 4.34% of the CMT and *S. aureus* positive bulk milk samples using PCR and ELISA, respectively, while the enterotoxin C gene was found only in one sample (2.17%) by PCR and did not detect by ELISA. The result of enterotoxin D was lower than the finding obtained in Iran, where the authors confirmed the presence of enterotoxin D in 12.5% of the examined samples using ELISA. The enterotoxin C finding came in agreement with a previous study in Egypt, in which the author confirmed the enterotoxin C in only one sample and failed to identify other enterotoxins in the same sample [34,35]. Our finding came in agreement with the previous result [36] and was higher than the result reported by Abd El-Tawab *et al*. [37] where the authors reported *sed* gene in 36.36% of raw milk samples. In this study, the ELISA test could not identify other classical enterotoxins in subclinical mastitis milk positive for *S. aureus* and this result agreed with Inganas *et al*. [38], Fey *et al*. [39] who confirmed that sandwich ELISA is the best available method for SE detection. However, the presence of protein A produced by *S. aureus* may be interfered with its sensitivity because of Protein A, which binds with Fe portion of the antibodies (IgG), causing false-negative results. The occurrence of both SEC and SED enterotoxins in *S. aureus* isolated from milk samples confirmed that these strains responsible for subclinical mastitis are from the animal origin where these enterotoxins are dominant if compared with other classical enterotoxins [40]. Furthermore, SED is produced in foods under a wider range of pH, redox potential (E_h_), and water activity (a_w_) than other SEs, which explain why SED is principal toxins involved in staphylococcal food poisoning [41,42].

Other virulence factors present in the *S. aureus*, including extracellular thermostable nuclease (*nuc*), toxic shock syndrome gene (*tsst*), and ­methicillin- restraint *Staphylococcus* (*mecA*) were investigated using PCR. Only the extracellular thermostable nuclease (*nuc*) gene was detected and confirmed in 71.73% of the examined milk samples confirmed positive for *S. aureus*. These findings were in fair agreement with the previous study [43] carried out in Egypt and confirmed the presence of *nuc* gene in all *S. aureus* strains, while, the (*tsst)* gene was confirmed in only one sample and they detected only the *mecA* gene in 53% of the examined samples [12]. Moreover, some phenotypically *S. aureus* strains were shown negative results for *nuc* gene, probably due to non-optimal experimental conditions for PCR method. The differences in the nucleotide sequence among the *nuc* genes were caused by some mutation or the absence of *nuc* gene in some *S. aureus* strains [44]. The results of this study confirmed no *S. aureus* MRSA strains. It concluded that *S. aureus* responsible for subclinical mastitis in this study mainly comes from animal origin rather than human ones according to Kitai *et al*. [45] who suggested that the source of food contamination by MRSA is mainly not of animal origin but from human as the food handlers and not from animal infection.

## Conclusion

*S. aureus* is one of the most important mastitis pathogens; it is a potential pathogen in cases of subclinical mastitis and considered one of the common public health hazards. The molecular assay is recommended for confirmation of the presence of *S. aureus* infection and its enterotoxin, especially enterotoxin D and extracellular thermostable nuclease (*nuc*) genes, which are the most common enterotoxin and virulence factors detected in this study. Human consumption of subclinical mastitis milk with the above-mentioned bacteria and its enterotoxins may lead to milk-borne intoxication, especially in children and old aged people. Therefore, CMT test is recommended to use as a screening test for the periodical examination of the dairy animals for the early detection of subclinical mastitis to help in rapid and effective treatment and prevention of this disease and avoid its spreading between dairy animals. In addition, the molecular and ELISA techniques are important to detect the different virulence factors carried in *S. aureus*. Moreover, the application of pre- and post-milking disinfection that will ensure the quality of milk and reflected on human health is required.

## Authors’ Contributions

RME and AATA designed the experiments, conducted the lab works, then drafted and edited the manuscript. Both authors read and approved the final manuscript.

## References

[1] Miller R.H, Paape M.J, Fulton L.A, Schutz M.M (1993). The relationship of somatic cell count to milk yields for Holstein heifers after first calving. J. Dairy. Sci.

[2] Seleim R.S, Rashed Y.M, Fahmy B.G.A (2002). Mastitis pathogens attachment-related virulence features, whey protein markers and antibiotic efficacy in cows. Vet. Med. J. Giza.

[3] Barker A.R, Schrick F.N, Lewis M.J, Dowlen H.H, Oliver S.P (1998). Influence of clinical mastitis during early lactation on reproductive performance of Jersy cows. J. Dairy. Sci.

[4] Sayed M, Rady A.A (2008). Acute clinically mastitic animals in villages of Assiut governance:Diagnosis and treatment. Vet. World.

[5] Letertre C, Perelle S, Dilasser F, Fach P (2003). Identification of a new putative enterotoxin SEU encoded by the egc cluster of *Staphylococcus aureus*. J Appl. Microbiol.

[6] European Food Safety Authority (2014) The European Union Summary Report on Trends and Source of Zoonoses, Zoonotic Agents and Food-borne Outbreaks in 2012 EFSA J.

[7] Fagundes H, Oliveira C.A.F (2004). *Staphylococcus aureus* intramammary infections and its implications in public health. Cienc. Rural.

[8] Vishnupriya S, Anthony P.X, Mukhopadhyay H.K, Pillai R.M, Thanislass J, Vivek S.V.M (2014). Methicillin-resistant staphylococci associated with bovine mastitis and their zoonotic importance. Vet. World.

[9] Jahan M, Rahman M, Parvej S, Chowdhury Z.H, Haque E, Talukder A.K, Ahmed S (2015). Isolation and characterization of *Staphylococcus aureus* from raw cow milk in Bangladesh. J. Adv. Vet. Anim. Res.

[10] Ewida R.M (2009). Some Studies on *Staphylococcus aureusa* in Milk and Some Milk Products Sold in Assiut City with Special Reference to Antibiotics Resistant *Staphylococcus aureus*.

[11] ElShall S.M (2019). Enterotoxins and Enterotoxigenic Staphylococci in Milk and Some Milk Products.

[12] Al-Ashmawy M.A, Sallam K.I, Abd-Elghany S.M, Elhadidy M.M, Tamura T (2016). Prevalence, molecular characterization, and antimicrobial susceptibility of methicillin-resistant *Staphylococcus aureus*. Foodborne Pathog. Dis.

[13] Baines J, Malek J, Speake G (2000). Cultural Atlas of Ancient Egypt. Checkmark Books.

[14] Radostitis O, Gay C, Blood D.C, Hinchcliff K.W (2006). Veterinary Medicine:A Textbook of the Diseases of Cattle, Horses, Sheep, Pigs and Goats.

[15] National Mastitis Council (1999). Laboratory and Field Handbook on Bovine Mastitis.

[16] Monday S.R, Bohach G.A (1999). Use of multiplex PCR to detect classical and newly described pyrogenic toxin genes in staphylococcal isolates. J. Clin. Microbiol.

[17] Løvseth A, Loncarevic S, Berdal K.G (2004). Modified multiplex PCR method for detection of pyrogenic exotoxin genes in staphylococcal isolates. J. Clin. Microbiol.

[18] Bayles K.W, Iandolo J.J Genetic and molecular analyses of the gene encoding staphylococcal enterotoxin. D. J. Bacteriol.

[19] Couch J.L, Soltis M.T, Betley M.J (1988). Cloning and nucleotide sequence of the type E staphylococcal enterotoxin gene. J. Bacteriol.

[20] Kuźma K, Malinowski E.H, Kłossowska A (2003). Specific detection of *Staphylococcus aureus* By PCR in intramammary infection. Bull. Vet. Inst. Pulawy.

[21] Murakami K, Minamide W, Wada K, Nakamura E, Teraoka H, Watanabe S (1991). Identification of methicillin-resistant strains of staphylococci by polymerase chain reaction. J Clin. Microbiol.

[22] Thrusfield M (2007). Veterinary Epidemiology.

[23] Schalm O.W, Noorlander D.O (1957). Experiments and observations leading to development of the California mastitis test. JAVMA.

[24] Elhaig M.M, Selim A (2015). Molecular and bacteriological investigation of subclinical mastitis caused by *Staphylococcus aureus* and *Streptococcus agalactiae* in domestic bovids from Ismalia, Egypt. Trop. Anim. Health Prod.

[25] Iraguha B, Hamudikuwanda H, Mushonga B, Kandiwa E, Mpatswenumugabo J.P (2017). Comparison of cow-side diagnostic tests for subclinical mastitis of dairy cows in Musanze district, Rwanda. J. South Afr.

[26] Reddy B.S.S, Kumari K.N, Reddy Y.R, Reddy M.V.B, Reddy B.S (2014). Comparison of different diagnostic tests in subclinical mastitis in dairy cattle. Int. J. Vet. Sci.

[27] Hundera S, Ademe Z, Sintayehu A (2005). Dairy cattle mastitis in and around Sebeta Ethiopia Intern. J. Appl. Vet. Med.

[28] Mekonnen H, Workineh S, Bayleyegne M, Moges A, Tadele K (2005). Antimicrobial susceptibility profile of mastitis isolates from cows in three major Ethiopian dairies. Med. Vet.

[29] Sadek O.A (2008). Human Health Risks Associated with Consumption of Milk from Subclinical Mastitic Animals in Assiut Governorate. Ph. D. Thesis. Assiut University.

[30] El-Gendy A.M (2015). Bacteriological and Molecular Studies on *Staphylococcus* Species Isolated from Raw Milk.

[31] Kamal R.M, Bayoumi M.A, Abd ElAal S.F.A (2013). MRSA detection in raw milk, some dairy products and hands of dairy workers in Egypt, a mini-survey. Food Control.

[32] Adwan G, Isayed H (2018). Prevalence and characterization of *Staphylococcus aureus* isolated from bulk tank milk dairy cow farms in West Bank-Palestine. Microbiol. Res. J. Int.

[33] Jones G.M, Bailey T.L, Roberson J.R (1998). Staphylococcus aureus mastitis:Cause, detection and control.

[34] Rahimi E, Mommtaz H, Shakerian A, Kavyani H.R (2012). The detection of classical enterotoxins of *Staphylococcus aureus* in raw cow milk using the ELISA method. Turk J. Vet . Anim. Sci.

[35] El-Baradei G.H (1998). The productivity of enterotoxins by *Staphylococcus aureus* strains isolated from mastitic milk and dairy handlers. Alex. J. Agric. Sci.

[36] Rola J.G, Korpysa-Dzirba W, Czubkowska A, Osek I (2015). Prevalence staphylococci recovered from raw cow milk. J. Dairy Sci.

[37] Abd El-Tawab A.A, Ammar A.M, El-Hafy F.I, Aideia H.A, Hammad E.A (2016). Bacteriological and molecular studies on toxigenic *Staphylococcus aureus* in milk and some milk products. BVMJ.

[38] Inganas M, Johansson S.G.O, Bennich H.H (1980). Interaction of human polyclonal IgE and IgG from different species with protein A from *Staphylococcus aureus*:Demonstration of protein-a-reactive sites located in the Fab2 fragment of human IgG. Scand. J. Immunol.

[39] Fey H, Pfister H, Ruegg O (1984). Comparative evaluation of different enzyme-linked immunosorbent assay systems for the detection of staphylococcal enterotoxins A, B, C, and D. J. Clin. Microbiol.

[40] Ahmed A.A.H, Studies on the Enterotoxigenicity of *Saureus* Isolated from Milk and Milk Products (1980). Ph. D. Thesis. Assiut University.

[41] Hunt K, Butler F, Jordan K (2014). Factors affecting staphylococcal enterotoxin C bovine production in milk. Int. Dairy J.

[42] Rajkovic A, Caballero B, Finglas P.M, Toldra F (2016). *Staphlococcus*:Food poisoning. Encyclopedia of Food and Health.

[43] Abd El-Hamid M, Bendary M.M (2013). Association between agr alleles and toxin gene profiles of *Saureus* isolates from human and animal sources in Egypt. Int. J. Adv. Res.

[44] Sahebnasagh R, Saderi H, Owlia P (2014). The prevalence of resistance to methicillin in *Staphylococcus aureus* strains isolated from patients by PCR method for detection of mecA and nuc genes. Iran. J. Public Health.

[45] Kitai S, Shimizu A, Kawano J, Sato E, Nakano C, Uji T, Kitagawa H (2005). Characterization of methicillin-resistant *Staphylococcus aureus* isolated from retail raw chicken meat in Japan. J. Vet. Med. Sci.

